# The Development of a Web-Based Tobacco Tracker Tool to Crowdsource Campus Environmental Reports for Smoke and Tobacco–Free College Policies: Mixed Methods Study

**DOI:** 10.2196/26280

**Published:** 2021-10-29

**Authors:** Sabrina F Loureiro, Kim Pulvers, Melissa M Gosdin, Keavagh Clift, Myra Rice, Elisa K Tong

**Affiliations:** 1 Center for Healthcare Policy and Research University of California Davis Sacramento, CA United States; 2 Department of Psychology California State University San Marcos San Marcos, CA United States; 3 Department of Occupational Health Services University of California Davis Sacramento, CA United States; 4 Department of Internal Medicine University of California Davis Sacramento, CA United States

**Keywords:** tobacco cessation, college smoke and tobacco–free policies, crowdsourcing, environmental reporting, public health, smoke and tobacco research

## Abstract

**Background:**

College campuses in the United States have begun implementing smoke and tobacco–free policies to discourage the use of tobacco. Smoke and tobacco–free policies, however, are contingent upon effective policy enforcement.

**Objective:**

This study aimed to develop an empirically derived web-based tracking tool (Tracker) for crowdsourcing campus environmental reports of tobacco use and waste to support smoke and tobacco–free college policies.

**Methods:**

An exploratory sequential mixed methods approach was utilized to inform the development and evaluation of Tracker. In October 2018, three focus groups across 2 California universities were conducted and themes were analyzed, guiding Tracker development. After 1 year of implementation, users were asked in April 2020 to complete a survey about their experience.

**Results:**

In the focus groups, two major themes emerged: barriers and facilitators to tool utilization. Further Tracker development was guided by focus group input to address these barriers (eg, information, policing, and logistical concerns) and facilitators (eg, environmental motivators and positive reinforcement). Amongst 1163 Tracker reports, those who completed the user survey (n=316) reported that the top motivations for using the tool had been having a cleaner environment (212/316, 79%) and health concerns (185/316, 69%).

**Conclusions:**

Environmental concerns, a motivator that emerged in focus groups, shaped Tracker’s development and was cited by the majority of users surveyed as a top motivator for utilization.

## Introduction

Smoke and tobacco–free (STF) policies among college and university campuses have become a high priority nationally to denormalize the use of tobacco [1,2]. The average smoker begins before the age of 26 years, making the transition into university life a significant period during which young people can either begin or avoid tobacco use [3]. Benefits of STF policies include decreased tobacco use and exposure to second-hand smoke, reduced tobacco litter, and greater well-being [4-6]. However, the benefits are contingent upon effective policy implementation and enforcement. Only 15% of US colleges detail methods for reporting an STF policy violation and over a third do not formally reprimand individuals who smoke on campus [7].

Low STF policy compliance is widespread at universities and poses a critical barrier to reducing tobacco use and related disease risk for the estimated 19.9 million college students in the United States [8-10]. The majority (62%) of colleges rely on social enforcement of the policy, with typical methods including education and outreach [7]. Given the responsibility placed with campus community members for enforcement, innovative approaches to engage them with the STF policy are needed [11]. One innovative approach, crowdsourcing, is a method of involving a large group of people on a collective mission and is being used to address a number of environmental and public health problems [12,13]. A crowdsourcing tool for tobacco activity surveillance can help address a major barrier to social enforcement: discomfort confronting tobacco users [7,14,15]. Further, it provides a sustainable solution to an infrastructure issue faced by most campuses lacking resources to collect data on campus tobacco use and related litter.

The goal of this study is to develop and implement a web-based tracking tool (Tobacco Tracker hereafter “Tracker”) to engage users with STF policies. This study aims to (1) describe the empirically driven process of developing and implementing Tracker using feedback from focus groups and (2) evaluate survey feedback from Tracker users about their motivations and experience using the tool.

## Methods

### Methods Overview

An exploratory sequential mixed methods approach was utilized to develop Tracker with guidance from campus focus groups and to evaluate Tracker with a user survey.

### Study Setting

The 2 universities in the study are located in northern and southern California. University 1, in northern California, has been STF since 2014. Its campus has over 39,000 students, 21,000 staff, and 2000 faculty, and its campus encompass 5300 acres including a separate health campus. University 2, located in southern California, has been STF since 2017, has over 14,000 students, over 300 administrative staff, and 600 academic staff, and its campus encompasses 304 acres. Approval from human subjects was obtained through the institutional review board at each university.

### Exploratory Focus Groups for Tracker Development

Focus groups aimed to explore ideas for prototype refinement were conducted in October 2018. A focus group comprising students was conducted at each university (n=7 at University 1 and n=10 at University 2). An additional focus group comprising employees was conducted at University 1 given its larger campus and staff size (n=6). Participants at University 1 were recruited from student or staff health and wellness listservs and a general staff electronic newsletter. Participants were selected in the order they responded to the recruitment advertisements and were offered a US $40 gift card. Participants at University 2 opted into the study from a web-based research portal and received credit toward their course requirement for participation. Eligibility included being at least 18 years old and a current university student or employee. Written informed consent was obtained from each participant prior to study participation. Each focus group lasted approximately 90 minutes.

To explore ideas for the Tracker prototype’s content and design, guiding questions for the focus groups explored the (1) appearance and functionality of the tool prototype, (2) communication channels and strategies for promoting the tool, and (3) images and messages that may raise awareness or increase motivation to use the tool. Educational information was presented from the California Tobacco Control Program’s public advertisements about how cigarette butts are the top litter problem and are not biodegradable with their plastic filters. All focus groups were conducted by the same moderator, a psychologist and tobacco control researcher (KP), audio recorded, and transcribed verbatim.

Two independent researchers, one of whom is a sociologist and qualitative researcher (MMG), conducted a thematic analysis [16]. Emergent codes were developed from the data inductively in addition to deductive priori codes. After the initial analysis, a codebook was constructed to create categories and develop themes. Two themes and 5 subthemes were developed collaboratively between the 2 independent researchers. No software programs were used to aid the focus group qualitative data analysis.

### Tracker Development

The Tracker prototype’s content, design, and promotion was refined on the basis of focus group findings, and themes linked to refined Tracker components are described below. Prototype components such as real-time web-based reporting and using visual maps to geographically locate environmental incidents were based on an existing crowdsourcing tool to measure energy overconsumption at University 1, TherMOOstat [17]. Tracker development in regard to content and design began in November 2018 and was promoted at both universities beginning in February 2019.

### Survey of Tracker Users

Individuals who used Tracker at either university between February 2019 and February 2020 and provided contact information for entry into an opportunity drawing were invited by email to participate in a survey in April 2020. A 2-week period was provided for a response and 1 reminder email was sent. An opportunity drawing for 4 US $25 gift cards at each university was offered. Survey questions developed by the study team assessed user experience: (1) how participants accessed the tool, (2) motivation for using the tool, (3) barriers to tool use and for future study directions, and (4) ideal actions following a report. Survey questions allowed for multiple responses and data were coded as a selection, no selection, or as missing. Data were reported as n (%) values in total and by university. 

## Results

### Focus Group Discussions

Focus group analysis yielded 2 distinct themes: (1) barriers and (2) facilitators to tool utilization (Table 1). Barriers to tool utilization contained 3 subthemes: logistical barriers limit tool use, information barriers, and framing as a policing tool. Facilitators for tool use contained 2 subthemes: environmental motivators and positive reinforcement to further engage with the tool. Minimal differences in responses were noted among the student participants, and differences between staff and student responses were minor (eg, less familiar with technology and less preference for a mascot or gamifying the tool). The results of thematic analysis are presented as an aggregate, given the goal of creating a tool for students and staff at both universities.

**Table 1:** Themes, subthemes, and representative quotes.

Themes, subthemes, and context				Representative quotes
**Theme: Barriers to tool utilization**				
	**Subtheme: Logistical barriers limit tool use**			
		Access to Tracker with QR^a^ codes	“What is it?” (University 1) “I’m in IT and we mock them” (University 1) “That’s kind of scary. I don’t know, that’s crazy. I’ve never done that.” (University 1)	
		Access to Tracker with campus app	“I think the campus app would just be the best solution, because a lot of [inaudible] have it installed already, but I think a lot of people have it installed. Rather than going and getting a whole new account or having to go find it somewhere else, it’s just an update and it’s easy” (University 2) “I really prefer the app a lot more… It seems more accessible in a sense. Like the online survey, you sometimes can’t access it or it doesn’t work. Obviously, that happens with apps too. It’s just more reliable.” (University 2)	
		Identifying location with automated GIS^b^ location services	“Yeah, it’s really useful, because you can find yourself. At least to me, it seems kind of like a combination of the dropdown and the map, because it’s just our campus and it has all the names of the buildings” (University 2) “Sometimes you’re like not at a building, you’re in between. But I do think sometimes people feel weird about sharing their locations.” (University 1)	
		Identifying location with a dropdown menu and a campus map	“It would be best to have both options available in the tool” (University 1) “I can see what you mean about the challenge of having it as a dropdown menu just from this example where you’re like ‘where’s my place’, it would be easier to type” (University 1)	
		Internet connection	“I feel like this might be going too out of the scope of this. The internet connection on campus is awful, and if it’s really slow, then I would just give up and not do it. That many reloading of pages would be really agonizing.” (University 1)	
	**Subtheme: Informational barriers**			
		Tobacco products	“What’s vaping?” (University 1) “I don’t know what Juul or blunts means” (University 1)	
		Tobacco Waste	“Are vaping things becoming garbage?” (University 1) “What is litter from vaping?” (University 2)	
		Image preference	“Just because it’s more identifiable because I’m on the go and I’m reporting this, I need to just see an actual picture.” (University 1) “It would be more helpful to have the different images of what you’re actually saying” (University 1)	
	**Subtheme: Framing as a policing tool**			
			“It also avoids the policing idea” (University 1) “It just likes a very, it’s an activity that’s very much in a specific location, especially since it’s forbidden to do it on campus, though. Again, I don’t feel it’s threatening, I don’t feel like it’s about policing people but rather about seeing signs of this activity. I can relate it to fire, but it could also be a consequence of smoking” (University 1) “I just think maybe a title that plays along with being environmentally conscious, instead of ‘Oh, I’m reporting somebody doing something bad and illegal,’ it’s more like, ‘I’m taking care of the environment.’ So it’s less of like a whistle-blower thing” (University 1) “Yeah. Because it almost seems like a tattling” (University 1)	
**Theme: Facilitators to tool utilization**				
	**Subtheme: Environmental motivators**			
		Design	“I think you should have a blue theme… That kind of ties in the blue sky” (University 1) “Well you don’t necessarily think of the trees, so I think that, and making the art with it, I think that was very effective. Because I never thought about trees before” (University 1) “You could have a bird with like a cigarette butt in its beak, like a cartoony bird. And maybe there could be like a friend bird with a fish in its beak, or something, next to it. I don’t know. But make sure they’re really cute” (University 1) “I said the squirrel with the [University 1] shirt and binoculars, because I think it directly ties into the community on campus, and squirrels are really [University 1]. And I think it’s good to have a cute little icon like the Thermoostat has because I think people want to use things that are cute, so I think it’s just good to have a main character” (University 1) “I said [University 1 mascot] Clean just because I think there’s the school pride, and then the clean is, I don’t know. Even with the air, I feel like that all goes back to like… I like that it’s hinting at the environment, but it’s still hinting at the smoking aspect versus the littering” (University 1)	
		Environmental concerns	“Yeah, simple, but I also learned something. I did not know it took up to a decade to decompose. That would make me want to report that” (University 1)	
	**Subtheme: Positive reinforcement**			
		Incentives	“I think if you provide incentives, people would definitely be up for that” (University 2) “Because I think it goes beyond this, and it’s more like why we recycle right, because it makes us feel good. Right I kind of see this tool as the same thing” (University 1)	
		Gamification	“Gamification is the way you’re going to win this” (University 1) “I think like also with stuff like this that’s so widespread, it can be hard to see any impact and nobody wants to do something, but it’s kind of hard to like want to be super engaged and do something all the time if you can’t really tell what you’re doing. I don’t know. Like making kind of like mini games or something where it’s like you’ve reported five times, and every time you report, you’re cleaning up lungs or something” (University 2)	

^a^QR: quick response.

^b^GIS: geographic information system.

### Barriers to Tracker utilization

#### Logistical Barriers

Participants identified logistical barriers to tool utilization, such as technical and access barriers. Participants cited the utilization of a dropdown menu and geographic information system (GIS) location–enabling services as potential technical barriers to tool utilization. Participants had mixed feelings about the utility of an automated GIS location–enabling service compared to the utilization of a dropdown menu of buildings from a campus map. Some individuals were reluctant to share their location with Tracker, while others felt it was more efficient and convenient. Participants concluded they would like both choices.

Participants also discussed various access barriers such as quick response (QR) codes, mobile apps, and internet connectivity. Some participant concerns about QR codes, a barcode that can be photographed with a phone to redirect a user to a webpage, included how to use them or negative attitudes about their utility (Table 1). Furthermore, participants discussed the use of mobile apps instead of accessing the tool through a website. However, some participants believed students would be less inclined to download another app that would take up storage space. Therefore, one recommendation was to integrate Tracker with an existing campus tool. Lastly, 1 participant expressed concerns that slow internet connectivity for devices or phones themselves could discourage the use of Tracker.

#### Informational Barriers

To identify tobacco use and waste, many participants expressed concern that they were unaware of what vaping products or vaping waste looked like. Participants recommended presenting visual examples of such tobacco products and waste (Table 1). Furthermore, participants preferred that these images look realistic rather than being a cartoon drawing.

#### Framing as a Policing Tool

Participants preferred the name and branding strategies to portray a neutral crowdsourcing tool. Specifically, participants were concerned about whether Tracker’s name would be viewed as public surveillance by the campus community’s smokers and nonsmokers. One participant suggested the use of an environmentally themed name to avoid portraying Tracker as one for policing fellow students and staff (Table 1). The participants preferred to de-emphasize the idea of policing smokers on campus.

### Facilitators for Tracker Utilization

#### Environmental Motivators

When participants were asked about Tracker’s design, environmentally themed color schemes such as blue or green and a name that suggested “clean” were preferred. Branding Tracker with an animal mascot, such as a dog or squirrel, was also recommended. Moreover, when presented with the California Tobacco Control Program’s public advertisements, participants found information about tobacco waste’s impact on the environment as a highly motivational reason to use Tracker.

#### Positive Reinforcement

To encourage continued use of Tracker, participants were asked about possible incentives. Participants agreed that incentives such as gift cards would encourage Tracker utilization. Some participants remarked that simply submitting a report would be rewarding enough for them to stay engaged. One user compared submitting a report to recycling and explained that the 2 actions made the individual feel motivated enough to continue.

To increase engagement with Tracker, student participants recommended that it be gamified to provide positive affirmation for Tracker reports on campus. Participants recommended that Tracker show a mascot dancing or interacting with the user for increased gratification and utilization. Other suggestions included using a reward system for reports to allow dressing the mascots.

### Empirically Driven Tracker Development

**Figure 1:**
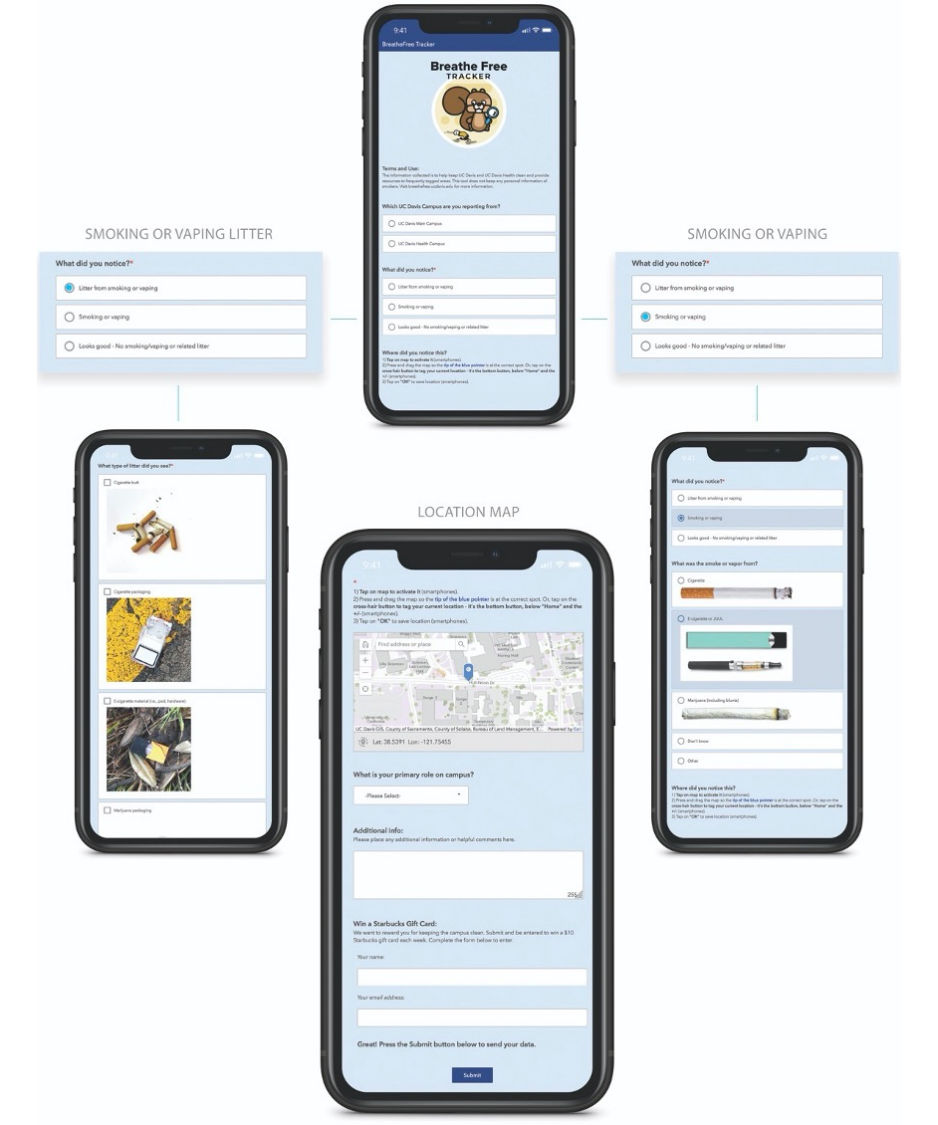
Images of the final Tracker tool and key components.

Based on focus group discussions, Tracker was designed for users to document tobacco use (smoking and vaping) or waste. Additionally, “no smoking or vaping” and “looks good” options were added for Tracker to have the capacity to reflect environmental improvements (Figure 1). Realistic images of tobacco products and waste were provided, in response to subtheme *Information Barriers*. Tracker instructions were framed to document observations of tobacco products and waste, not people, and the word “report” was purposefully avoided; instead, users were asked “what do you notice” and “where did you notice this” in response to subtheme *Framing as Policing Tool* as a barrier. Tracker was branded with environmentally themed names affiliated with the STF programs on each campus in response to subtheme *Environmental Motivator*. A squirrel mascot was adopted at University 1 and a hawk mascot at University 2, keeping with the environmental design theme.

The study team created a Tracker website for each university with features responsive to subtheme *Logistical Barriers*. The website-based platform Esri Survey 123 supported accessibility and subsequent integration with existing campus technological infrastructure, such as the campus app and websites. While both universities have a personal campus website account, mostly used by students, only University 1 allowed integration with Tracker. Multiple options for location tracking were provided to users: automatic GIS-enabled mobile location services, manual designation on the campus map, or manual selection of a building location from a drop-down menu. Additionally, graphic designers helped design images or promotional messages. Promotion consisted of flyers or advertisements and items (eg, stickers and bags) distributed at campus events.

Incentives for positive reinforcement, in response to subtheme *Positive Reinforcement* were also incorporated in Tracker. A positive affirmation message after the user pushed the submit button states, “Great! Your data was sent successfully. Thanks.” To provide a reinforcing feedback loop, the message further stated, “Feedback is used to identify areas of concern and inform future policy efforts” where users may click on a link to a live map of submitted reports. The “Looks good. No smoking/vaping or related litter” option to report problem-free areas created an additional opportunity to build positive reinforcement in messages emphasizing a clean campus. Additionally, a weekly gift card raffle incentive was offered. The study team decided that gamification would need further development for future efforts.

### Survey of Tracker Users

In total, 1163 discreet responses were documented in total from both universities’ Tracker, and 717 individuals who provided an email ID were invited to participate in the user survey (n=605 users at University 1 and n=112 users at University 2). Tracker user survey responses by university are shown below (Table 2). The response rate for University 1 was 40% (242/605 invitations) and that for University 2 was 70% (78/112 invitations). Four duplicate surveys were omitted (University 2), yielding a total of 316 survey responses. Over half of the survey participants at both universities reported accessing Tracker through a website. Approximately a quarter of respondents at University 2 also reported using a QR code or campus app; however, few participants (10 of 242 participants, <5%) at University 1 reported using these methods.

**Table 2:** Tracker user survey results (N=316).

			Total, n (%)^a^		University 1 (n=242), n (%)		University 2 (n=74), n (%)
**How did you access the Tracker?**							
	Personal campus website portal	N/A^b^		102^c^ (45.9)		N/A	
	STF^d^ or Tracker-specific website	148 (54.4)		114 (51.4)		34 (68.0)	
	Campus app	21 (7.7)		7 (3.2)		14 (28.0)	
	QR^e^ code	18 (6.6)		5 (2.3)		13 (26.0)	
	Other	11 (4.0)		10 (4.5)		1 (2.0)	
**What motivates you to use the Tracker?**							
	A clean environment is important to me	212 (78.8)		172 (78.2)		40 (81.6)	
	Exposure to smoke or vapor is harmful to health	185 (68.8)		152 (69.1)		33 (67.3)	
	Smoking/vaping on campus encourages others to smoke/vape	110 (40.9)		87 (39.5)		23 (46.9)	
	I support the campus smoke and tobacco free policy	181 (67.3)		148 (67.3)		33 (67.3)	
	I believe campus policies should be followed	137 (50.9)		108 (49.1)		29 (59.2)	
	Smoking/vaping does not fit the campus image	87 (32.3)		72 (32.7)		15 (30.6)	
	Using the Tracker gives me an opportunity to win a gift card	98 (36.4)		82 (37.3)		16 (32.7)	
**Which of the following might discourage you from using the Tracker?**							
	I intend to use the Tracker, but forget	131 (50.6)		111 (51.9)		20 (44.4)	
	It takes too long to access the tool	108 (41.7)		87 (40.7)		21 (46.7)	
	There are too many questions	46 (17.8)		36 (16.8)		10 (22.2)	
	The instructions are not clear	23 (8.9)		19 (8.9)		4 (8.9)	
	The smoking/vaping or litter does not bother me very much	2 (0.8)		2 (0.9)		0	
	I am concerned people would know I reported	31 (12.0)		25 (11.7)		6 (13.3)	
	I do not want people to get in trouble	20 (7.7)		18 (8.4)		2 (4.4)	
	I feel uncomfortable using the Tracker	8 (3.1)		7 (3.3)		1 (2.2)	
	Other	34 (13.1)		31 (14.5)		3 (6.7)	
**Ideally, what would you like to happen after you report smoking/vaping or related litter?**							
	The litter will be cleaned up	205 (77.9)		171 (78.8)		34 (73.9)	
	The violator will be assigned community service	117 (44.5)		98 (45.2)		19 (41.3)	
	The violator will be fined	109 (41.4)		94 (43.3)		15 (32.6)	
	The violator will be assigned a diversion training	70 (26.6)		58 (26.7)		12 (26.1)	
	A new sign will be installed	69 (26.2)		61 (28.1)		8 (17.4)	
	The violator will be sanctioned	49 (18.6)		42 (19.4)		7 (15.2)	
	Other	24 (9.1)		19 (8.8)		5 (10.9)	

^a^Total number of responses differed between questions and universities. In response to “How did you access the Tracker”, a total of 272 responses were recorded (222 at University 1; 50 at University 2); “What motivates you to use the Tracker”, a total of 269 responses (220 at University 1; 49 at University 2); “Which of the following might discourage you from using the Tracker” a total of 259 responses (214 at University 1; 45 at University 2); and “Ideally, what would you like to happen after you report smoking/ vaping or related litter?” a total of 263 responses (217 at University 1; 46 at University 2).

^b^N/A: not applicable.

^c^Percent sums over 100% are due to select-all type responses.

^d^STF: smoke and tobacco–free.

^e^QR: quick response.

The top motivations to use Tracker included environmental concerns, health concerns, and policy support. The majority (212/269, 78.8%) cited the importance of a clean environment, 185 (69%) participants were concerned about health hazards of exposure to smoke or vapor, and 181 (67%) voiced personal support for the campus STF policy.Approximately half (137/269, 50.9%) of the participants also expressed personal support that campus policies should be followed, and less than half (110/269, 40.9%) reported concerns that tobacco use encouraged others to smoke. Approximately one-third of participants (98/269, 36.4%) reported being motivated by campus images or the opportunity to win a gift card.

In regard to utilization barriers to Tracker, logistical issues were the leading barriers at both universities: of 259 participants, 131 (50.6%) intended but forgot to use Tracker, 108 (41.7%) were discouraged because it took too long to access Tracker, and 46 (17.8%) thought that Tracker had too many questions.Information barriers were infrequently cited, and few reported that the instructions were unclear or that they were unbothered by the tobacco use or waste. Other infrequently cited responses included concerns about a policing tool, concerns that others would know about their reporting, not wanting others to get in trouble, and being uncomfortable using Tracker.

When asked about users’ ideal outcome following a Tracker report, the majority (205/263, 77.9%) of participants across both universities voiced environmental concerns with having the litter cleaned up. There was also interest in the consequences for policy violators including community service (117/263, 44.5%) and fines (109/263, 41.4%), followed by diversion trainings (70/263, 26.6%). Additional signage installation was selected by one-fourth (69/263) of respondents.

## Discussion

### Principal Findings

To our knowledge, this is the first reported study to describe the development of an empirically derived web-based Tracker for crowdsourcing campus environmental reports of tobacco use and waste to support STF college policy compliance. Campus focus group discussions identified barriers and facilitators for Tracker utilization, which informed Tracker development. Over 1 year, Tracker at both universities had been used by over 1000 people. In response to the survey, Tracker users cited environmental concerns as the leading motivator for utilization, complementary to focus group findings, followed by health concerns and STF policy support. Tracker users also cited having litter cleaned up as the ideal outcome, further highlighting their environmental concerns. Most potential barriers identified in the focus groups were not cited by Tracker users, although refinements should still be considered.

Health concerns from environmental and second-hand smoke/vapor, cited by Tracker users as top motivators for using Tracker, is consistent with broader population concerns, which may be due in part to existing public health educational campaigns. Environmental motives are consistent with a national study of 8000 students, which revealed that the majority (66%) reported that a college’s commitment to environmental issues contributed to their decision to apply or attend a school [18]. Instead of focusing on individual smokers’ behaviors and beliefs, which can create defensiveness, focusing on extrinsic issues, such as environmental concerns and second-hand smoke, has been recommended to promote a more receptive audience [19]. Public health education campaigns about the hazards of tobacco waste and second-hand smoke/vapor exposure are already found in the national Truth Campaign and state campaigns through the California Department of Public Health’s Tobacco Free California [20,21] Future efforts incorporating environmentally focused public health campaign messages on campus could activate and reinforce crowdsourcing participation with Tracker.

Tracker was designed to minimize accessibility issues. The use of a web-based platform appears to have been a good choice, as accessing Tracker though campus websites was more common than through the Campus App or from QR codes. However, users cited the leading barrier as the time it took to access Tracker. Further study is needed to understand and resolve this issue. For example, it could be a convenience issue, such that users may be too focused on their commute to stop and access Tracker. This could also be related to Tracker users’ second most frequently reported barrier: “intended to use the Tracker but forgot.” Additional user research could inform modifications to make accessing Tracker faster and more efficient.

Positive reinforcers were built into the Tracker design to drive activation, crowdsourcing participation, and STF policy engagement. In response to focus group suggestions to provide a positive affirmation message, the Tracker message after a report submission stated its intended goal: to identify areas of concern and inform policy efforts. However, the user survey finding that the top desired action following the submission of a report was to “clean up litter.” The positive affirmation messaging could be refined for greater specificity. For example, the message could provide more tangible feedback, such as “Your report has contributed to the clean-up of x number of tobacco litter items on campus.” Interestingly, the opportunity to win a gift card was one of the least common reasons for using Tracker, suggesting that this incentive was not a strong reinforcement and could be scaled back or removed.

Our findings show mixed support for formal enforcement of Tracker. Focus group participants expressed concerns about policing, whereas Tracker users reported some preference for consequences for policy violators with community service or fines. Although punitive approaches such as fines may be perceived as a solution, they could decrease perceptions of university support among those who smoke [22]. A combined approach, including social and formal enforcement, may be warranted [9]. For example, the information gathered through crowdsourcing can be used to complement existing campus outreach programs, such as peer-led Ambassador programs, to target outreach or clean-up efforts in areas of concentrated tobacco activity [23]. For colleges without the resources for an ambassador or comparable enforcement program, the Tobacco Tracker provides a surveillance mechanism to monitor tobacco activity on campus and demonstrate to campus stakeholders the need for intervention [24].

There is growing interest in using crowdsourcing tools to effect change for the environment, including tracking tobacco use and litter. Ohio State’s “Cleaner U” app focuses on all types of litter on their college campus [25] instead of just smoke and tobacco litter, similar to Tracker. Meanwhile, the World Health Organization’s “Tobacco Spotter” app focuses on reporting compliance or noncompliance with a broad array of tobacco control policies in different countries, spanning tobacco retail, advertisement, and nonsmoking public policies [26]. Lastly, “Litterati,” a smartphone app aimed at encouraging users to pick up litter in their respective communities, maps litter reports, including smoke and tobacco litter. Litterati’s user community cleaned up approximately 50,000 pieces of litter within 1 week [27]. While these tools have effectively used crowdsourcing to report environmental issues, Tobacco Tracker uses crowdsourcing to record environmental reports of STF policies, namely tobacco use and waste, on college campuses.

### Limitations

Our results must be interpreted with caution, owing to certain limitations. While both universities studied herein represent public systems with 100% STF policies, only 1 university per system was included, and the results may not be generalizable to those of other universities or outside of California. Furthermore, focus group sizes were small and participant-level characteristics were not assessed in the focus groups or user surveys. Therefore, it is not known how representative the respondents are of the university populations. Furthermore, Tracker user experience is limited to those who provided an email and responded to the survey and may not represent others, including those who decided not to use Tracker.

### Conclusions

Environmental concerns successfully shaped Tracker’s development and were commonly cited as the top motivators of use. Educating the campus community about the environmental hazards of tobacco waste may increase support for STF policies, especially among young adults [28]. Tracker is a promising tool that addresses environmental and health concerns to help support STF college policy compliance. Using Tracker, the campus community can be engaged to uphold STF policies by crowdsourcing data to help monitor campus areas in need of improvement. Future efforts may include refining Tracker for increased user accessibility or engagement and to target outreach and clean-up efforts. Additional research is needed to measure Tracker’s effect on improving compliance with STF campus policies.

## Abbreviations

GIS: geographic information system
QR: quick response
STF: smoke and tobacco–free
